# Social inequalities in indicators of use of healthcare services by adolescents in Campinas, São Paulo, Brazil

**DOI:** 10.1590/0102-311XEN146523

**Published:** 2024-04-29

**Authors:** Vivian Castro Lemos, Marilisa Berti de Azevedo Barros, Margareth Guimarães Lima

**Affiliations:** 1 Faculdade de Ciências Médicas, Universidade Estadual de Campinas, Campinas, Brasil.

**Keywords:** Social Inequalities, Medical Care, Adolescent Health Services, Health Survey, Desigualdades Sociais, Cuidados Médicos, Serviços de Saúde do Adolescente, Inquérito de Saúde, Desigualdades Sociales, Atención Médica, Servicios de Salud del Adolescente, Encuesta de Salud

## Abstract

This study aimed to analyze the prevalence of indicators of use of healthcare services according to sex, income and race/skin color, in adolescents (aged 10-19 years old) based on data from the *Health Survey of the Municipality of Campinas* (ISACamp), carried out in 2014/2015 in Campinas, São Paulo, Brazil. The chi-square test was used to evaluate the differences between the outcome variables (indicators of use of healthcare service) and sex, income and race/skin color. Adjusted prevalence ratios (PR) were estimated using Poisson multiple regression models. The demand for medical care was high in the last year of the interview (79.2%), mostly attended by the Brazilian Unified National Health System (65.2%), with routine consultations being more prevalent for females (PR = 1.17; 95%CI: 1.01-1.34) and injury for the male population (PR = 0.47; 95%CI: 0.26-0.84). Economic and racial differences were found in the evaluation of the last medical consultation, with a higher prevalence of worse care among those with lower income (PR = 1.46; 95%CI: 1.14-1.87) and black people (PR = 1.27; 95%CI: 1.01-1.61). Inequalities remained for delay or failure to carry out exams (PR = 1.64; 95%CI: 1.02-2.64) and worse quality of dental care (PR = 2.10; 95%CI: 1.38-3.21) in those with lower income. Also, black people had fewer appointments with dentists (PR = 0.90; 95%CI: 0.82-0.99).

## Resumo

O estudo objetivou estimar a prevalência dos indicadores de uso de serviços de saúde de acordo com sexo, renda e etnia/cor da pele entre adolescentes (10 a 19 anos) com base nos dados do *Inquérito de Saúde no Município de Campinas* (ISACamp), realizada em 2014/2015 em Campinas, São Paulo, Brasil. O teste qui-quadrado foi utilizado para avaliar as diferenças entre as variáveis de desfecho (indicadores de utilização de serviços de saúde) e sexo, renda e etnia/cor da pele. As razões de prevalência (RP) ajustadas foram estimadas por meio de modelos de regressão múltipla de Poisson. A demanda por atendimentos médicos foi elevada no último ano da entrevista (79,2%), atendidos majoritariamente pelo Sistema Único de Saúde (65,2%), sendo as consultas de rotina mais prevalentes no sexo feminino (RP = 1,17; IC95%: 1,01-1,34) e lesão na população masculina (RP = 0,47; IC95%: 0,26-0,84). Diferenças econômicas e raciais foram encontradas na avaliação da última consulta médica, com maior prevalência de pior atendimento entre aqueles com menor renda (RP = 1,46; IC95%: 1,14-1,87) e negros (RP = 1,27; IC95%: 1,01-1,61). Desigualdades permaneceram em termos de atraso ou falha na realização de exames (RP = 1,64; IC95%: 1,02-2,64) e pior qualidade do atendimento odontológico (RP = 2,10; IC95%: 1,38-3,21) naqueles com menor renda. E os negros consultaram menos os serviços de dentistas (RP = 0,90; IC95%: 0,82-0,99).

## Resumen

El objetivo de este estudio fue estimar la prevalencia de los indicadores de uso de servicios de salud según el sexo, ingresos y etnia/color de la piel entre adolescentes (10 a 19 años) basado en los datos de la *Encuesta de Salud de la Ciudad de Campinas* (ISACamp), realizada entre 2014 y 2015 en Campinas, São Paulo, Brasil. Se utilizó la prueba de chi-cuadrado para evaluar las diferencias entre las variables de resultado (indicadores de uso de servicios de salud) y sexo, ingresos y etnia/color de la piel. Se estimaron las razones de prevalencia (RP) ajustadas a través de modelos de regresión múltiple de Poisson. La demanda de atención médica fue alta en el último año de la entrevista (el 79,2%), mayoritariamente asistidos por el Sistema Único de Salud (el 65,2%), con las consultas de rutina más frecuentes para el sexo femenino (RP = 1,17; IC95%: 1,01-1,34) y la atención por lesiones más frecuentes para la población masculina (RP = 0,47; IC95%: 0,26-0,84). Se encontraron diferencias económicas y raciales en la evaluación de la última consulta médica, con mayor prevalencia de peor atención entre aquellos con menores ingresos (RP = 1,46; IC95%: 1,14-1,87) y personas negras (RP = 1,27; IC95%: 1,01-1,61). Las desigualdades persistieron en términos de retraso o no realización de exámenes (RP = 1,64; IC95%: 1,02-2,64) y peor calidad de la atención odontológica (RP = 2,10; IC95%: 1,38-3,21) en aquellos con menores ingresos. Y las personas negras fueron las que menos consultaron los servicios de dentistas (RP = 0,90; IC95%: 0,82-0,99).

## Introduction

Treating the health of adolescents holistically attenuates possible adverse health conditions in adulthood, avoiding undesirable complications stemming from poor medical care and attendance ^1^. Fostering health promotion in adolescents enables reaching the phase of maturity with quality of life, as health demands are very specific in this phase, involving physical, emotional and hormonal transformations, which can be monitored by healthcare services ^2^ ^,^ ^3^.

Seeking healthcare may be less valued in adolescence than in other periods of life, leading to the nonadherence to treatment and preventive care. Seeking health services is linked to individual perceptions of one’s own body and situations of health and illness. Sociocultural-, geographic-, and transportation-related aspects as well as the offer and organization of healthcare systems are related to scenarios of possible difficulties in accessing medical care and other healthcare services ^4^ ^,^ ^5^ ^,^ ^6^. A survey conducted in the city of Pelotas (Brazil), in 2012, found that only 23% of the adolescents interviewed reported having used healthcare services ^4^.

Problems with the reception and humanization of medical consultations also exert a negative influence on the frequency of use and access to healthcare services, which is unfavorable to the creation of bonds between adolescents and healthcare staff, hindering interactive dialog and active listening. This has an impact on the satisfaction and wellbeing of each user of the system ^7^. Some publications from the Brazilian Ministry of Health provide detailed information for health professionals, addressing the specificities of healthy development and illness in these youths. The Adolescent Health Booklet is a primary care support tool that enables the recording of pertinent data for clinical appointments ^1^.

The vulnerabilities of this population should also be considered. In 2015, the Brazilian Institute of Geography and Statistics (IBGE, acronym in Portuguese) reported that 53.2% of individuals among black and brown populations, aged from 18 to 24 years were still in primary school and high school, whereas this figure was 29.1% among white individuals. This context holds a great influence on the unequal distribution of wealth in Brazil, as 76% of the 10% poorest individuals were black and brown; in contrast, 22.8% were white. The inequality perpetuated against vulnerable populations - including setbacks in the educational system - affects the parents and guardians of children and adolescents, consequently also affecting these young people ^8^ ^,^ ^9^.

Epidemiological studies have shown an increase in records of morbidity and mortality related to external causes as a result of exposure to situations of risk and violence ^10^. One study analyzing 30 years of the burden of disease in Brazil found that at least 50,000 adolescents and young people die of avoidable causes - such as interpersonal violence, injuries, suicide, and accidents in both sexes but three times more among males - every year. Such occurrences tend to be greater among subgroups situated on a lower socioeconomic level ^11^.

Social issues, accentuated income inequalities, and racial discrimination found in Brazil significantly impact the population’s health and also affect the search for healthcare services ^12^ ^,^ ^13^ ^,^ ^14^ ^,^ ^15^. The *Brazilian National Survey of School Health* (PeNSE, acronym in Portuguese) conducted in 2015 and 2019 demonstrated that the search for health services and professionals was more frequent among female and white adolescents as well as students who attended private schools ^12^ ^,^ ^13^.

Healthcare for adolescents occurs through the use of services. Understanding the form of access, frequency and reasons for seeking healthcare services is a fundamental strategy for expanding public policies directed at this age group and circumventing the notion that individuals at this age are always healthy ^1^ ^,^ ^2^. However, there are few studies addressing this topic in Brazil, especially those with an analysis of the impact of socioeconomic inequalities ^4^ ^,^ ^10^ ^,^ ^12^ ^,^ ^13^ ^,^ ^16^ ^,^ ^17^. Therefore, this study aimed to investigate the prevalence of indicators of use of healthcare services according to sex, monthly family income per capita and race/skin color in the adolescent population (10-19 years) living in the municipality of Campinas, which is the second largest city in the state of São Paulo.

## Methods

A population-based, cross-sectional study was conducted using data from the third health survey conducted in the city of Campinas in 2014 and 2015. For such, 3,021 individuals of both sexes were interviewed, with the random selection of independent samples for the following age groups: 10-19 years, 20-59 years, and 60 years or older.

A total of 70 census sectors were selected (14 sectors from each of the five administrative districts of the city: east, northwest, north, southwest, and west), which constituted the strata. These sectors were selected based on the definition established in the 2010 *Demographic Census*.

A list was compiled in the field to confirm the existence of the households in these 70 sectors and update addresses. The selection of households per sector was then carried out systematically, beginning with the sectors and followed by residences, considering a 20% of losses. To reach this representative sample, 2,898 households were selected for adolescents, 950 for adults and 3,326 for older people with the aim of approaching the margin of 1,000 individuals for each age group. This number considered 50% proportions of indicators (maximum variability of the sample), an alpha of 5% and a deff of 2. Greater detailing on the sampling process has been published previously ^18^.

All individuals in the age group of interest were interviewed in the households selected for the adolescent population. This study analyzed data on individuals aged 10-19 years.

The questionnaire had 12 blocks of questions related to diseases and complaints, accidents and violence, emotional health, health and wellbeing, use of services, preventive practices, immunization, use of medications, and socioeconomic characteristics. The questionnaire was administered by trained interviewers and the data were recorded on electronic devices (tablet computers). Participation was authorized by the parents or guardians of minors by signing a statement of informed consent. After this signature, the adolescents were invited to participate while respecting their autonomy. Interviews were held with the adolescents themselves.

The outcomes of the study were indicators of the use of health services. The first question aimed to measure the use of healthcare services and access to medical care by investigating the occurrence of medical appointments in the previous year. The answers were categorized as “no” (0) (appointment more than one year earlier) or “yes” (1) (appointment within the previous year). The following aspects were also investigated: reason for the last appointment in the previous year or more than one year earlier (disease or health problem, injury or routine checkup); assessment of the care received, with answers grouped as “good” (0) or fair/poor/very poor (1); coverage by private health insurance (0) or the Brazilian Unified National Health System (SUS, acronym in Portuguese) (1); and whether the respondent felt well oriented to take care of the problem [“yes” (0) or “no” (1)]. The following variables were analyzed to assess access to and the use of health services for specific health conditions: had problems or was unable to obtain a medical appointment in the previous year [“no” (0) or “yes” (1)]; delay or the non-performance of exams requested during the appointment [“no” (0) or “yes” (1)]; delay or unable to schedule an appointment with a specialist [“no” (0) or “yes” (1)]; and visited a dentist in the previous year [“no” (0) (visited a dentist more than one year earlier) or “yes” (1) (visited a dentist within the previous year)]. The assessment of the last dental appointment [good/very good (0) or fair/poor/very poor (1)] and whether the respondent had ever been vaccinated for hepatitis B [“no” (0) or “yes” (1)] were also investigated.

The independent variables were sex [male (0) or female (1)], family income per capita [≥ the monthly minimum wage (0) or < the monthly minimum wage (1)], self-declared race/skin color [white (0) and black (1)]; the latter category was composed of black and brown individuals as proposed by the IBGE and the Brazilian National Health Policy for the Black Population ^8^ ^,^ ^9^. Other categories (Asian descent and Indigenous) were not considered in this study due to the lack of information.

Starting from this set, a descriptive table was structured to guide the investigations carried out in this study, covering the variables, questions and respective answers (Box 1).

**Box 1:** Description of variables of use of healthcare services.

OUTCOME VARIABLE	QUESTION ASKED	ANSWER
Medical appointment in previous year (G101)	When was the last time you had an appointment with a physician?	(0) The last appointment was more than one year ago (1) The last appointment was in the previous year
Main reason for the appointment (G103)	What was the main reason you had to schedule your last appointment with a physician?	(0) Disease/Health problem (1) Injury (2) Routine checkup
Assessment of attendance of the last appointment as fair/poor/very poor (G117)	How do you assess the care received at the last medical appointment?	(0) Good, very good (1) Fair, poor, very poor
Coverage of appointment through the SUS (G108)	Who covered or complemented the cost of this appointment?	(0) Company plan, individual health insurance plan, other (1) SUS
Felt well oriented to take care of problem (G118)	Did you feel well oriented and informed about how to take care of your problem?	(0) No (1) Yes
Had health problem for which was unable to obtain an appointment in previous year (G119A)	Have you had any health problem for which you were unable to obtain an appointment in the last year?	(0) No (1) Yes
There was delay or non-performance of exams requested during medical appointment (G120A and G121A)	Was any exam requested during a medical appointment in the last year that took a long time for you to get the results? Was any exam requested during a medical appointment in the last year that was not performed? These two questions were combined for the outcome YES	(0) No (1) Yes
There was delay or was not able to schedule an appointment with a specialist physician (G122A and G123A)	Was any referral to a specialist or other health professional requested during any medical appointment in the last year that took a long time to be scheduled? Was any referral to a specialist or other health professional requestion during any medical appointment in the last year that you were unable to schedule? These two questions were united for the outcome YES	(0) No (1) Yes
Dental appointment in previous year	When was the last time you visited a dentist?	(0) Last appointment was more than one year before (1) Last appointment was in the previous year
Assessment of dental appointment as fair/poor/very poor	How do you assess the dental care that you received?	(0) Good, very good (1) Fair, poor, very poor
Has been vaccinated against hepatitis B	Have you ever had a vaccine against hepatitis B?	(0) No (1) Yes
EXPOSURE VARIABLES		ANSWER
Sex		(0) Male (1) Female
Monthly family income per capita		(0) < monthly minimum wage (1) ≥ monthly minimum wage
Race/Skin color		(0) White (1) Black (black/brown)

SUS: Brazilian Unified National Health System.

Statistical analysis consisted of calculations of the prevalence of the outcomes with respective 95% confidence intervals (95%CI). Comparisons with the independent variables were performed using the chi-square test with the Rao-Scott correction. Multiple Poisson regression models with robust variance were run to estimate prevalence ratios (PR) and respective 95%CI, with one model run for each outcome. To control for confounding factors, the analyses stratified by sex were adjusted by age; the analyses stratified by skin color were adjusted by sex, age, and income; and the analyses stratified by income were adjusted by sex, age, and skin color. Due to the complex survey sampling methods, the Stata 15.0 program (https://www.stata.com) was used, which considers weights of non-responses, post-stratification and study design ^18^.

The 2014/2015 survey was approved by the Human Research Ethics Committee of the School of Medical Sciences at the State University of Campinas (certificate n. 409,714/2013; the present project was approved under certificate n. 5,283,905/2022).

## Results

A total of 1,022 adolescents aged 10-19 years were interviewed, which was 10.4% lower than the target sample. The 10-to-14-year-old and 15-to-19-year-old age groups accounted for 48.4% and 51.6% of the sample, respectively. Girls accounted for 49.1% of the sample and boys accounted for 50.9%. Blacks (black and brown) accounted for 43.7% of the sample and white individuals accounted for 56.3%. Among the income strata, 59.4% of the adolescents had a family income per capita of less than the monthly minimum wage and 2.7% had a family income per capita of more than three minimum wages (data not shown).

Table 1 shows that 79.2% of the adolescents living in Campinas visited a physician in the previous year and 21.3% assessed the appointment as fair, poor, or very poor. Most individuals were attended at the SUS and 85.2% felt well oriented to take care of their problem. Only 8.1% of the population of adolescents reported being unable to obtain an appointment for some health problem. A total of 66.9% had dental appointments and 11.7% of these appointments were assessed as fair, poor, or very poor. A total of 97.3% of the population aged 10-19 years (Table 1) was vaccinated against hepatitis B.

**Table 1:** Prevalence and prevalence ratios of indicators of use of healthcare service among adolescents (10-19 years) according to sex. Campinas, São Paulo, Brazil, 2014/2015.

Variables	Total [% (n)]	Male * [% (95%CI)]	Female [% (95%CI)]	PR (95% CI) **
Medical appointment in the previous year	79.2 (803)	76.4 (71.9-80.3)	82.1 (78.5-85.2)	1.08 (1.01-1.15)
Main reason for last appointment				
Disease/Health problem	45.1 (458)	46.6 (41.1-52.2)	43.5 (38.2-48.8)	0.93 (0.81-1.06)
Routine checkup	43.4 (438)	40.2 (34.5-46.1)	46.7 (40.9-52.5)	1.17 (1.01-1.34)
Injury	5.3 (52)	7.1 (5.3-9.5)	3.3 (2.1-5.4)	0.47 (0.26-0.84)
Assessment of attendance of the last appointment as fair/poor/very poor	21.3 (219)	19.4 (16.2-23.1)	23.2 (19.4-27.5)	1.19 (0.99-1.43)
Coverage of appointment through the SUS	65.2 (654)	65.0 (58.9-70.6)	65.3 (58.9-71.3)	1.00 (0.92-1.09)
Felt well oriented to take care of problem	85.2 (856)	86.2 (82.5-89.2)	84.1 (80.0-87.5)	0.98 (0.93-1.02)
Problem for which was unable to obtain an appointment in previous year	8.1 (84)	6.1 (4.4-8.6)	10.1 (7.8-13.0)	1.65 (1.11-2.45)
There was delay or non-performance of exams requested during medical appointment	7.2 (77)	5.1 (3.4-7.7)	9.4 (6.8-12.9)	1.81 (1.04-3.15)
There was delay or was not able to schedule an appointment with specialist physician	8.9 (87)	7.1 (5.1-9.9)	10.9 (8.1-14.4)	1.54 (1.07-2.22)
Dental appointment in previous year	66.9 (678)	67.4 (62.0-72.4)	66.3 (59.6-72.4)	0.98 (0.89-1.09)
Assessment of dental appointment as fair/poor/very poor	11.7 (111)	9.5 (7.2-12.5)	13.9 (10.1-18.8)	1.45 (1.01-2.11)
Has been vaccinated against hepatitis B	97.3 (925)	96.7 (94.1-98.2)	97.8 (95.0-99.1)	1.01 (0.99-1.03)

95%CI: 95% confidence interval; PR: prevalence ratio; SUS: Brazilian Unified National Health System.

* Reference: male sex;

** Regression model run for each indicator of healthcare service use adjusted by age.

Table 1 also shows associations between indicators of use of healthcare services and sex. Females reported more frequent medical appointments in the previous year (PR = 1.08; 95%CI: 1.01-1.15), appointments for routine checkups (PR = 1.17; 95%CI: 1.01-1.34), not being able to schedule an appointment for some health problem in the previous year (PR = 1.65; 95%CI: 1.11-2.45), faced some delay or were not able to perform the requested exams (PR = 1.81; 95%CI: 1.04-3.15) and faced some delay or was not able to schedule an appointment with a specialist physician (PR = 1.54; 95%CI: 1.07-2.22) and assessed dental care as poor (PR = 1.45; 95%CI: 1.01-2.11). Appointments due to injuries were more frequent among male adolescents (PR = 0.47; 95%CI: 0.26-0.84) (Table 1).

Regarding household income per capita, those who reported less than the monthly minimum wage (lower stratum) had fewer routine medical appointments in the previous year (PR = 0.92; 95%CI: 0.85-0.98) as well as a fewer appointments for a routine checkup (PR = 0.82; 95%CI: 0.73-0.94) compared to those with a higher income. This population also had lower frequencies of receiving proper orientation to take care of the health problem (PR = 0.94; 95%CI: 0.90-0.99) and had fewer dental appointments (PR = 0.79; 95%CI: 0.71-0.89). Furthermore, adolescents identified in the lower income stratum used services offered by the SUS more often (PR = 1.59; 95%CI: 1.38-1.82), but reported difficulties in scheduling exams (PR = 1.64; 95%CI: 1.02-2.64), with higher frequencies of assessing medical and dental appointments as fair, poor, or very poor (PR = 1.46; 95%CI: 1.14-1.87 and PR = 2.10; 95%CI: 1.38-3.21, respectively) (Table 2).

**Table 2:** Prevalence and prevalence ratios of health service use indicators among adolescents (10-19 years) according to income. Campinas, São Paulo, Brazil, 2014/2015.

Variables	n	Income *		PR (95%CI) **	PR (95%CI) ***
		≥ Monthly minimum wage [% (95%CI)]	< Monthly minimum wage ^#^ [% (95%CI)]		
Medical appointment in the previous year	800	82.6 (78.8-85.9)	76.7 (72.8-80.3)	0.92 (0.86-0.98)	0.92 (0.85-0.98)
Main reason for last appointment					
Disease/Health problem	457	41.5 (34.9-48.4)	47.6 (43.0-52.2)	1.16 (0.99-1.36)	1.16 (0.98-1.36)
Routine checkup	437	48.2 (40.8-55.8)	40.1 (36.0-44.3)	0.81 (0.71-0.93)	0.82 (0.73-0.94)
Injury	52	4.7 (3.2-6.8)	5.7 (4.1-7.9)	1.26 (0.76-2.08)	1.32 (0.79-2.20)
Assessment of attendance of the last appointment as fair/poor/very poor	219	16.5 (13.4-20.3)	24.7 (20.7-29.0)	1.51 (1.19-1.93)	1.46 (1.14-1.87)
Coverage of appointment through the SUS	654	46.3 (39.8-52.9)	77.9 (72.8-82.3)	1.69 (1.46-1.95)	1.59 (1.38-1.82)
Felt well oriented to take care of problem	854	88.1 (84.2-87.9)	83.3 (79.5-86.5)	0.94 (0.89-0.99)	0.94 (0.90-0.99)
Problem for which was unable to obtain an appointment in previous year	84	6.4 (4.6-8.8)	9.3 (7.0-12.1)	1.42 (0.93-2.16)	1.31 (0.82-2.09)
There was delay or non-performance of exams requested during medical appointment	77	4.9 (3.1-7.6)	8.9 (7.0-11.3)	1.84 (1.16-2.92)	1.64 (1.02-2.64)
There was delay or was not able to schedule an appointment with specialist physician	86	7.1 (4.6-10.7)	10.0 (7.4-13.5)	1.38 (0.85-2.24)	1.45 (085-2.47)
Dental appointment in previous year	675	76.7 (71.6-81.2)	59.9 (53.9-65.7)	0.78 (0.69-0.87)	0.79 (0.71-0.89)
Assessment of dental appointment as fair/poor/very poor	111	6.8 (4.8-9.5)	15.4 (11.8-19.8)	2.25 (1.51-3.36)	2.10 (1.38-3.21)
Has been vaccinated against hepatitis B	923	96.3 (92.8-98.1)	97.9 (95.7-99.0)	1.01 (0.99-1.04)	1.01 (0.98-1.04)

95%CI: 95% confidence interval; PR: prevalence ratio; SUS: Brazilian Unified National Health System.

* Family income per capita;

** Regression model run for each indicator of healthcare service use adjusted by age and sex;

*** Adjusted by age, sex, and skin color;

^#^ Reference: income < monthly minimum wage.

Table 3 shows differences concerning race/skin color. After adjusting for sex, age and income, the assessment of medical appointments as fair/poor/very poor was significantly greater among black individuals compared to white ones (PR = 1.27; 95%CI: 1.01-1.61). Frequencies were also higher among black individuals for appointments in the SUS (PR = 1.27; 95%CI: 1.14-1.42), although these adolescents had visited a dentist less often (PR = 0.90; 95%CI: 0.82-0.99).

**Table 3:** Prevalence and prevalence ratios of health service use indicators among adolescents (10-19 years) according to race/color. Campinas, São Paulo, Brazil, 2014/2015.

Variables	n	Race/Skin color		PR (95%CI) *	PR (95%CI) **
		White *** [% (95%CI)]	Black [% (95%CI)]		
Medical appointment in the previous year	791	78.3 (74.7-81.5)	79.9 (75.4-83.7)	1.02 (0.96-1.09)	1.04(0.97-1.12)
Main reason for last appointment					
Disease/Health problem	452	43.9 (38.4-49.6)	46.5 (41.0-52.1)	1.05 (0.91-1.22)	1.03 (0.89-1.19)
Routine checkup	433	45.9 (39.5-52.5)	40.2 (35.2-45.4)	0.88 (0.75-1.03)	0.91 (0.77-1.07)
Injury	51	5.0 (3.5-7.2)	5.5 (3.8-7.8)	1.05 (0.63-1.75)	0.99 (0.59-1.66)
Assessment of attendance of the last appointment as fair/poor/very poor	215	18.3 (14.9-22.3)	25.0 (21.1-29.3)	1.38 (1.10-1.73)	1.27 (1.01-1.61)
Coverage of appointment through the SUS	646	55.5 (48.9-62.0)	77.8 (73.0-82.0)	1.40 (1.25-1.57)	1.27 (1.14-1.42)
Felt well oriented to take care of problem	844	85.3 (81.1-88.7)	84.7 (80.6-88.0)	0.99 (0.94-1.05)	1.01 (0.94-1.06)
Problem for which was unable to obtain an appointment in previous year	83	6.8 (5.0-9.3)	9.7 (7.2-13.0)	1.46 (0.94-2.25)	1.37 (0.85-2.2)
There was delay or non-performance of exams requested during medical appointment	76	5.5 (3.8-7.9)	9.5 (7.0-12.7)	1.76 (1.10-2.83)	1.60 (0.99-2.58)
There was delay or was not able to schedule an appointment with specialist physician	87	9.8 (7.3-12.9)	8.2 (5.4-12.2)	0.85 (0.53-1.36)	0.81 (0.48-1.35)
Dental appointment in previous year	673	71.7 (66.0-76.8)	61.6 (56.1-66.9)	0.86 (0.78-0.94)	0.90 (0.82-0.99)
Assessment of dental appointment as fair/poor/very poor	110	9.7 (7.1-13.2)	14.3 (11.3-18.1)	1.50 (1.10-2.06)	1.31 (0.92-1.86)
Has been vaccinated against hepatitis B	914	97.1 (94.0-98.6)	97.8 (95.5-99.0)	1.01 (0.98-1.03)	1.01 (0.98-1.03)

95%CI: 95% confidence interval; PR: prevalence ratio; SUS: Brazilian Unified National Health System.

* Regression model run for each indicator of health service use adjusted by age and sex;

** Adjusted by age, sex, and income per capita;

*** Reference: white individuals.

## Discussion

This study offers findings on the use of healthcare services by adolescents in the city of Campinas, addressing the main reasons for the use of services in this population, assessment of the care provided and public or private coverage. The study also offers information on the lack of or difficulty in accessing exams and treatment of specific health problems as well as information on vaccination for hepatitis B. These issues were analyzed according to sex, income, and skin color.

Our findings reveal a high frequency of medical appointments by adolescents in the year prior to the interview (79.2%), with females and individuals in the higher income category presenting more frequent use of healthcare services. Being ill and routine checkup were the main reasons for seeking healthcare. A total of 65.2% of the adolescent population of Campinas sought care in the public healthcare system and 85.2% felt well-oriented with regards to caring for their problem. This draws attention to the high demand on the public healthcare system and demonstrates success in the expanded offer of care.

In agreement with our findings on the considerable use of the public health care system among adolescents in the city of Campinas, the 2019 PeNSE found that 74.1% of students sought basic health units (BHU), which are services within the SUS network ^10^, and 52.1% of the sample of adolescents in a population-based study conducted in the city of Pelotas also scheduled appointments via the SUS, underscoring the scope and relevance of the Brazilian public healthcare system ^4^. The Brazilian School Health Program is an important intersectoral policy involving healthcare teams, such as the Family Health Strategy (FHS), and has contributed to expanding strategic health education actions in the public education system since its implantation in 2007 to stimulate and propagate health promotion and knowledge ^19^ ^,^ ^20^.

The use of healthcare services and its entire context are related to the needs of each individual and the extent to which signs and symptoms generate discomfort; the use of healthcare services also reflects the conditions of mobility and care offered in each geographic region ^6^ ^,^ ^21^ ^,^ ^22^. Studies have pointed out inequalities among different groups and sociodemographic factors related to the use of services in Brazil ^4^ ^,^ ^6^ ^,^ ^15^ ^,^ ^23^.

General medical appointments and those for routine checkups in the previous 12 months were more frequent among female adolescents. This greater predisposition to healthcare among females has been reported in studies involving different age groups, including adolescents, as reported in the 1998 *Brazilian National Household Sample Survey*, the survey conducted in Pelotas in 2012 as well as the PeNSE published in 2012, 2015, and 2019 ^4^ ^,^ ^12^ ^,^ ^13^ ^,^ ^16^ ^,^ ^23^ ^,^ ^24^.

This and other studies found that the use of health services is greater among those with a disease and/or health problems, which are determinants of access ^12^ ^,^ ^22^. On the other hand, four out of every 10 adolescents sought routine care, demonstrating proactive behavior with regards to self-care within the scope of prevention and health promotion offered by the FHS and BHU. The greater frequency of appointments among girls may be based on the Women’s Healthcare Policy, which is well established in the practice of the teams at health units, as well as the availability of gynecologists. The 2015 *Health Survey of the Municipality of Campinas* offered important observations on the greater number of early diagnoses of chronic diseases for females, which could be linked to the greater seeking of health services in adolescence, favoring greater control of these comorbidities throughout life ^25^.

Medical appointments due to injuries were found predominantly in males, revealing divergencies between the sexes at early ages. This finding agrees with data from the 2009 and 2015 PeNSE, which found an increasing trend of indicators of violence, such as involvement in fights with knives and firearms among male students ^26^. Exposure to situations of vulnerability is more likely to affect the male population; indeed, accidents and episodes of violence correspond to 40% of deaths in the 10-to-19-year-old age group in Brazil, predominantly affecting the male sex ^3^ ^,^ ^27^ ^,^ ^28^. This search for healthcare services mostly due to emergency situations could lead to premature illness by chronic diseases in this population, as it renders proper follow-up of healthy development unviable in this important phase of the lifecycle.

Only 8.1% of the adolescents reported being unable to obtain care for a specific problem in the previous year. However, analyses by group revealed some indicators of specific difficulties regarding access to healthcare services, such as not being able to schedule a medical appointment for a specific problem, delays, or the non-performance of exams, delays or not being able to schedule appointments with a specialist physician and poorer medical and dental care. These aspects were more commonly reported by females, which is related to the greater use of services by females in all age groups and the fact that females visit healthcare facilities more for themselves as well as for children and other family members. This could lead to clearer perceptions of the care received, reflecting social and cultural behaviors of that structure perspectives of gender in Brazilian society ^15^.

Regarding income, an evident pattern of inequalities was observed, as adolescents with a lower family income had lower frequencies of medical appointments, routine checkups, and dental appointments in the previous year and also assessed the quality of the care received as poor. During appointments, poor orientation to take care of the problem as well as delays or the non-performance of requested exams were more frequent in the lower income stratum. These results demonstrate how socioeconomic inequalities affect proper support and the offer of care free of prejudice, which are determinants of physical and mental health and contribute to the protection of human rights ^29^. A population-based study conducted in the city of Pelotas (2012) found greater use of healthcare services in the month prior to the interview by adolescents in income classes A and B, which are economically more privileged ^4^. Individuals with a higher income are more likely to have private health insurance plans, with greater access to the diagnoses of their chronic diseases, complaints, and symptoms.

In the analyses stratified by race/skin color, no associations were found with the use of healthcare services, measured by appointments in the year before the interview or by the reasons for seeking care (disease or health problem, routine checkup and injury). We also found no differences in orientations given to care for a specific problem. These outcomes demonstrate equity for white and black individuals in the city of Campinas with regards to several indicators.

Despite the greater equality in the use of health services according to race, the evaluation of medical services as being poor was more prevalent among black individuals, even after adjusting for income. These results indicate that, despite the good coverage of healthcare services for vulnerable populations, racial discrimination and segregation may still be negatively influencing the quality of care offered. Data from the 2013 *Brazilian National Health Survey* indicated that 10.6% of individuals older than 18 years had experiences of feeling discriminated or being treated worse than others at healthcare services, especially among black and brown individuals and those with less schooling, which is another indicator used to measure socioeconomic inequalities ^8^.

The obstacles are diverse and can be impacted by possible integration deficiencies in the healthcare network in a regionalized manner, inadequate staff at certain services (care voids), the heterogeneity of healthcare teams, some of which are engaged little and do not follow guidelines on proper reception and humanization of care, as well as incomplete primary care staff, which affects the coverage of the population, leading to dissatisfaction of users of the system with regards to the quality of medical and dental care ^13^ ^,^ ^30^.

Diseases treated since childhood and/or adolescence is fundamental to the clinical management of diagnoses, the determination of types of treatment, the offer of services and the autonomy of individuals to seek the integrated health network ^31^. Chronic health conditions can worsen considerably over one’s lifetime and often begin in early phases. The organization and planning of care for these morbidities in the beginning of life can subsequently lead to a better overall health ^32^.

A favorable scenario of the high use of health services and equity in several indicators according to income and race/skin color was found in this study and is in line with the universality of the SUS, ensuring the constitutional right to healthcare for the population in a broad and interconnected way and promoting the expansion of primary care. The Brazilian National Primary Care Policy and Brazilian National Health Promotion Policy implemented in 2006 and continually updated provide actions directed at prevention, health promotion and primary care, with primary care units constituting the essential gateway of users of the system and the maintenance of health protection ^30^. For some indicators, however, especially the assessment of the quality of the care received, disparities regarding income and race/skin color are still found, indicating the need for the maintenance and strengthening of these policies. The lack of or delay in access to exams was also more prevalent in the group with a lower socioeconomic status.

One of the strengths of this investigation was the focus on the adolescents’ health, which is an underexamined group in population-based studies, with the analysis of several indicators of the use of healthcare services to gain an understanding of the set of situations that encompass care and the quality of care offered. Another strength was the analysis of perceived inequalities affecting the most vulnerable (those with a low income and black skin color).

This study also holds limitations, such as the lack of comparisons of the results with data from previous studies on racial and economic topics in the adolescent population, mainly due to the scarcity of such studies in the literature. Information bias, especially recall bias, regarding reports of the use of and access to health services is another limitation, especially considering that 20.8% of the adolescents sought medical appointments more than one year before the interview.

There are numerous challenges in the field of adolescent care and it is necessary to improve communication between intersectoral administrations, such as health and education. However, population-based surveys aimed at clarifying frequencies and reasons for the use of health services, care coverage, etc. constitute powerful tools for strategic and more assertive planning. The recognition that adolescents must visit health services underscores the importance of offering quality healthcare, which implies the creation and reorganization of care models, especially directed at professional training and humanization, guiding health policies and practices for this public. However, social, economic and racial disparities remain in the quality of care offered, which violates human rights and such injustices can lead to severe consequences.
